# Recent Exploration of Solid Cancer Biomarkers Hidden Within Urine or Blood Exosomes That Provide Fundamental Information for Future Cancer Diagnostics

**DOI:** 10.3390/diagnostics15050628

**Published:** 2025-03-05

**Authors:** Tomoaki Hara, Sikun Meng, Aya Hasan Alshammari, Hideyuki Hatakeyama, Yasuko Arao, Yoshiko Saito, Kana Inoue, Eric di Luccio, Andrea Vecchione, Takaaki Hirotsu, Hideshi Ishii

**Affiliations:** 1 Department of Medical Data Science, Center of Medical Innovation and Translational Research, Osaka University Graduate School of Medicine, Suita, Yamadaoka 2-2, Osaka 565-0871, Japan; 2 Hirotsu Bio Science Inc., Chiyoda-Ku, Tokyo 102-0094, Japan; 3 Department of Clinical and Molecular Medicine, University of Rome “Sapienza”, Santo Andrea Hospital, Via di Grottarossa, 1035-00189 Rome, Italy

**Keywords:** cancer, biomarkers, diagnostics, exosome, RNA

## Abstract

Cancer cells exhibit abnormal behavior compared to normal cells. They ignore growth arrest signals such as contact inhibition, a mechanism that stops their proliferation when they collide with surrounding cells, and proliferate in an uncontrolled manner, destroying tissue. Early detection and treatment of cancer are therefore important for healthy longevity. Cancer cells differ from normal cells in their characteristic gene expression due to their abnormalities. Cancer markers that reflect these characteristics have been searched for and applied to diagnosis. Although analysis of blood antigens has been the main method, further development of a diagnostic system is needed for early detection of cancer. Next-generation sequencers have improved gene expression analysis technology, making it possible to analyze detailed gene expression in cancer cells and nucleic acid molecules in blood or urine. In addition, cancer cells release extracellular vesicles, exosomes, which are known to contain molecules that may serve as cancer markers. This review summarizes the latest findings on exosomal cancer markers.

## 1. Introduction

Cancer cells behave in a selfish manner and use every means at their disposal for their own proliferation [1,2,3]. They change energy metabolism, adapt to hypoxia by enhancing the glycolytic system, promote proliferation by activating cell cycle signaling pathways, form new blood vessels to supply nutrition, acquire the ability to metastasize to other organs via blood and lymph fluids, acquire immune evasion ability by expressing immune checkpoint molecules such as PD-L1, and develop cancer stem cells that show resistance to chemotherapy [4,5,6,7]. It is a difficult task to achieve complete cures for the large amounts of cancerous tissues that have acquired all of these abilities. Therefore, early detection and treatment of cancerous tissues are important in completely curing cancer [8]. For this purpose, diagnosis of cancer using cancer markers in blood and urine is recommended [9]. Therefore, a search for cancer markers that can accurately diagnose early-stage cancer is being conducted [10,11]. In particular, exosomes released by cancer cells contain many cancer-specific molecules and are expected to be applied to cancer diagnosis [12]. Exosomes are extracellular vesicles of about 30–150 nm in diameter, with CD9, CD63, and CD81 as common exosome markers on their surface and various RNAs and proteins inside [13]. Exosomes are released when the cell membrane is entrapped to form endosomes, vesicles are formed within the endosomes, and these endosomes fuse with the cell membrane. Exosomes can be separated from blood and urine by ultracentrifugation, immunoaffinity, size exclusion, and precipitation [14]. Exosomes derived from cancer cells have been found to be involved in cancer growth, metastasis, angiogenesis, and immune escape, and are released into the blood and urine [15]. This review summarizes recent reports on candidate cancer markers present in the exosomes of cancer patients.

## 2. Search for Exosome-Derived Cancer Markers in Blood

Cancer diagnosis using blood cancer markers has become common [16,17]. Exosomes derived from cancer are circulating in the blood [18]. Therefore, collection of exosomes from blood and analysis of their contents can lead to the identification of useful cancer markers (Table 1).

### 2.1. Blood Exosomes in Breast Cancer Patients

This section describes the latest reports on blood exosome markers for breast cancer. Exosomes from the blood of 120 breast cancer patients and 60 healthy controls were collected and small RNA sequencing identified about 3500 small RNAs. A diagnostic model was constructed with six small RNAs (piR-36,340, piR-33,161, miR-484, miR-548ah-5p, miR-4282, and miR-6853-3p), and the area under the curve (AUC) was 0.972. The targets of these microRNAs (miRNAs) were involved in the chemokine signaling pathway [19]. miR-200c was quantified in blood exosomes from 51 breast cancer patients and 47 healthy controls. miR-200c expression was downregulated in breast cancer patients. The AUC of miR-200c was 0.854, whereas those of CEA, CA125, and CA153 were 0.615, 0.700, and 0.727, respectively. In those four combined models, the AUC was 0.914, sensitivity was 91.49%, and specificity was 76.6% [20]. Exosome sequencing was performed on the blood of three chemotherapy-sensitive patients and three drug-resistant patients with triple-negative breast cancer (TNBC). A total of 85 miRNAs were identified whose expression varied in relation to chemotherapy resistance. In addition, when six miRNAs (miR-182, miR-1246, miR-378-e, miR-6730-3p, miR-6831-5p, and miR-373) were quantified by RT-qPCR in 30 TNBC patients, only miR-6831-5P showed expression fluctuation, indicating that miR-6831-5P was downregulated in drug-resistant patients [21]. An integrated centrifugal disk chip (CD chip) was developed for rapid quantification of surface proteins of blood exosomes, and target proteins could be quantified within 10 min with 100 μL of blood. Exosomes from breast cancer cells (MCF-7) and normal mammalian epithelial cells (MCF-10A) were collected and Western blotting was used to detect EpCAM, PSMA, HER2, EGFR, CEA, and CA125, and a marked increase in expression of EGFR, CEA, and CA125 was observed in MCF-7. EpCAM was detected in MCF-7 and MCF-10A to the same extent, and PSMA and HER2 were slightly upregulated in MCF-7. Quantification of CEA, CA125, and EGFR on the surface of blood exosomes of six breast cancer patients and two healthy controls with a CD chip was able to identify breast cancer patients [22]. Quantification of CD9- and HER2-positive exosomes from pre- and postoperative blood samples of eight breast cancer patients showed a trend toward lower exosome levels after surgery [23]. Blood exosomes from 30 breast cancer patients, 30 colorectal cancer patients, 30 lung cancer patients, and 39 healthy controls were collected and quantified for PD-L1, EpCAM, and EGFR, showing that the expression of these genes was increased in cancer patients. Diagnostic modeling with PD-L1, EpCAM, and EGFR showed that the AUC was 0.978, sensitivity was 90.0%, specificity was 97.4%, and accuracy was 94.2% for breast cancer, AUC was 0.977, sensitivity was 96.7%, specificity was 100%, and accuracy was 98.6% for colorectal cancer, and AUC was 0.936, sensitivity was 80.0%, and accuracy was 98.6% for lung cancer [24]. The above miRNAs and proteins are expected to be used as exosome markers in breast cancer blood for diagnosis.

### 2.2. Blood Exosomes of Lung Cancer Patients

This section describes the latest reports on blood exosome markers for lung cancer. Blood exosomes from 24 patients with squamous-cell lung cancers (SQCLCs), 24 patients with lung adenocarcinomas (LUADs), and 24 healthy controls were collected for miRNA analysis, and eight differentially expressed miRNAs (miR-21-5p, miR-126-3 p, miR-210-3p, miR-221-3p, Let-7b-5p, miR-146a-5p, miR-222-3p, and miR-9-5p) were detected. Diagnostic models were constructed with each miRNA, and diagnostic tests were performed, showing that the AUC for SQCLC was 0.646–0.740, sensitivity was 62.5–83.3, and specificity was 66.7–87.5, and for LUAD, the AUC was 0.535–0.655, sensitivity was 41.7–79.2, and specificity was 4.2–91.7 [25]. Blood exosomes from 31 non-small-cell lung cancer (NSCLC) patients were collected and miR-29a-3p was quantified by RT-qPCR. miR-29a-3p expression was upregulated in the 20 patients in the non-relapse group [26]. Blood exosomes from 305 NSCLC patients and 226 healthy controls were collected and snoRNAs were quantified by RT-qPCR to identify those with altered expression. Among them, SNORD116 and SNORA21 were downregulated in NSCLC patients, with AUCs of 0.738 and 0.775, respectively. When traditional blood biomarkers CYFRA21-1 and carcinoembryonic antigen (CEA) were combined with SNORD116 and SNORA21, the AUC was 0.917. Furthermore, this diagnostic model was able to identify 132 metastatic NSCLC patients and 173 non-metastatic NSCLC patients with an AUC of 0.784 [27]. Blood exosomes from nine lung cancer patients and three healthy controls were collected and quantified for mRNAs, long non-coding RNAs (lncRNAs), miRNAs, and circular RNAs (circRNAs) by microarray and RNA-seq, showing that 591 mRNAs, 881 lncRNAs, 45 miRNAs, and 916 circRNAs were upregulated, while 108 mRNAs, 135 lncRNAs, and 480 circRNAs were downregulated in lung cancer patients. RT-qPCR of circ-0033861, circ-0043273, and circ-0011959 showed that they were significantly upregulated in lung cancer patients [28]. Blood exosomes of 79 patients with or without lung cancer metastasis were collected and gene expression analysis identified five genes (CXCL12, TFBR2, CD44v6, HIF1A, and KRT7) important for diagnosing metastasis. A diagnostic model was constructed with these genes and the AUC was 0.9488 [29]. Exosomes from the blood of 30 NSCLC patients were collected and nested PCR was used to detect EGFR mutations, showing a sensitivity of 76.6% [30]. Exosomes from the peripheral blood of 10 small-cell lung cancer (SCLC) patients were collected from peripheral blood mononuclear cells (PBMC-EXs) and analyzed for proteins. Protein analysis revealed increased expression of c-Myc and Snail and decreased expression of MAVS and STING in chemoimmunotherapy-treated non-responder cells. Furthermore, when responder exosomes were fed to SCLC cell lines, apoptosis was observed to be more pronounced than in the case of non-responder cells [31]. Exosome spectra of lung cancer cell lines (NCI-H226, HCC-827, and A549) and normal cell line (BEAS-2B) were analyzed by surface-enhanced Raman spectroscopy (SERS) and a diagnostic model was constructed using machine learning. Blood exosomes from 20 lung cancer patients and 20 healthy controls were collected for diagnostic testing, showing that the AUC was 0.84, sensitivity was 83.3%, and specificity was 83.3% [32]. Blood exosomes from 60 lung cancer patients, 95 breast cancer patients, and 78 healthy controls were collected for detection of alpha-linked Thomsen–Friedenreich glycoantigen (TF-Ag-α). TF-Ag-α was detected in patients with cancer and the diagnosis could be made with more than 95% accuracy. This indicates that TF-Ag-α is a new exosomal carbohydrate biomarker [33]. As described above, RNAs and proteins have been identified as exosome markers in lung cancer blood. It is interesting to note that circular RNAs and small nucleolar RNAs (snRNAs) involved in RNA modification also have potential diagnostic applications.

### 2.3. Blood Exosomes of Pancreatic Cancer Patients

This section describes the latest reports on blood exosome markers for pancreatic cancer. Blood exosomes of five pancreatic cancer patients and five healthy controls were collected and lncRNAs with altered expression were identified by RNA-seq. Four of them (LINC01268, LINC02802, AC124854.1, and AL132657.1) were further quantified in 78 pancreatic cancer patients and 70 healthy controls by RT-qPCR. When the diagnostic efficacy of these four lncRNAs was analyzed, the AUC, sensitivity, and specificity were 0.8476, 0.72, and 0.89, respectively [34]. Exosomes from the blood of 10 pancreatic cancer patients were collected and miRNAs were analyzed by microarray. miR-6855-5p was found to be associated with radioresistance. Further analysis of 28 pancreatic cancer patients confirmed a trend toward increased radioresistance in the group with higher expression of blood exosome-derived miR-6855-5p [35]. Blood exosomes from 18 pancreatic cancer patients and 16 healthy controls were collected for detection of miR-21, miR-191, and miR-451a by molecular beacon–peptide (MBP) probes, showing increased expression of these miRNAs in pancreatic cancer patients [36]. miRNA analysis of blood exosomes collected from 20 pancreatic cancer patients by microarray revealed that six miRNAs (miR-10394-5p, miR-6779-5p, miR-197-5p, miR-4327, miR-638, and miR-12117) were involved in gemcitabine-based preoperative treatment responder and three miRNAs (miR-6891-5p, miR-6732-5p, and miR-1234-3p) were involved in the poor responder. A diagnostic model with these miRNAs was further constructed and diagnostic tests were performed on 66 patients, showing that the AUC was 0.777 for responders and 0.685 for poor responders [37]. Blood exosomal gene expression data from 164 pancreatic cancer patients and 118 healthy controls in ExoRBase 2.0. were analyzed with the LASSO Cox regression model, showing that expression of ARNTL2, FHL2, KRT19, MMP1, CDCA5, and KIF11 was associated with pancreatic cancer. The prognosis was worse in the ARNTL2, KRT19, MMP1, CDCA5, and KIF11 high-expression group and the FHL2 low-expression group [38]. The above miRNAs, lncRNAs, and proteins were identified as exosome markers in pancreatic cancer blood. It was interesting that lncRNAs were detected as markers and they are increasingly expected to be studied for diagnostic applications.

### 2.4. Blood Exosomes in Colorectal Cancer Patients

This section describes the latest reports on blood exosome markers for colorectal cancer. Blood exosomes from 58 colorectal cancer (CRC) patients and 28 healthy controls were collected and NAMPT-Antisense (NAMPT-AS) and Nicotinamide phosphoribosyl-transferase (NAMPT) mRNA were quantified by RT-qPCR, showing that they were upregulated in patients with CRC. Elevated expression of serum NAMPT was also observed in patients with CRC. Diagnostic models of each showed an AUC of 0.65 for NAMPT-AS, 0.646 for NAMPT mRNA, and 0.632 for serum NAMPT [39]. Blood exosomes from 31 T1a-stage CRC patients, including 22 colon cancer (CC) patients and 9 rectum cancer (RC) patients, 19 precancerous advanced adenoma (AA) patients, and 10 healthy controls were collected and subjected to RNA-seq analysis to identify genes with variable expression. A CRC prediction model was constructed with eight RNAs (Let-7f-5p, C19orf43, TOP1, PPDPF, lnc-MKRN2-42:1, LNC-EV-9572, HIST2H2AA4, and miR-320a-3p), and the AUC was 0.76. An AA prediction model was also constructed with nine RNAs (miR-425-5p, Let-7f- 5p, C19orf43, TOP1, PPDPF, LNC-EV-9572, lnc-MKRN2-42:1, HIST2H2AA4, and MT-ND2), and the AUC was 0.88 [40]. Blood exosomes from six CRC patients and four healthy controls were collected and 5-methylcytosine miRNA-21 (m5C-miRNA-21) was quantified by DNAzyme-RCA-based colorimetric and lateral flow dipstick assays, showing that m5C-miRNA-21 was upregulated in patients with CRC [41]. Proteomic analysis of blood exosomes collected from 25 CRC patients and 12 healthy controls by mass spectrometry identified about 60 proteins with variable expression. A diagnostic model constructed using machine learning with ELISA data from 912 patients revealed that PF4 and AACT, which were upregulated in CRC patients, were important for CRC diagnosis, with an AUC of 0.963 in the diagnostic test [42]. The above miRNAs, lncRNAs, and proteins were identified as exosome markers in breast cancer blood, and the fact that RNA methylation was detected as a marker is intriguing. The application of the RNA modification perspective to diagnosis is increasingly expected.

### 2.5. Blood Exosomes in Other Cancer Patients

This section describes the latest reports on blood exosome markers for cancers other than those described in the previous sections. Analysis of blood RNA data (GSE164174) and gene expression data (TCGA) in gastric cancer (GC) identified four miRNAs (miR-21-5p, miR-320, miR-191-5p, and miR-451) for diagnosing metastasis. A diagnostic model was constructed for metastasis with these miRNAs and found that the AUC was 0.86, which is higher than that of the existing markers, CEA, CA19-9, CA125, and CA72-4 [43]. An SERS sensor was developed for exosome retrieval and target protein detection. When the MUC1 protein was quantified in blood exosomes of 15 gastric cancer patients and 5 healthy subjects, a significant increase in MUC1 expression was observed in gastric cancer patients [44]. When a diagnostic model was constructed using blood exosome gene expression data of hepatocellular carcinoma (HCC) patients extracted from exoRBase, RNA-seq data from cancer tissues from the TCGA database, and scRNA-seq data from the GEO database, the AUC was 0.847 in the exoRBase HCC cohort [45]. Blood exosomes from nine HCC patients were collected and proteomics analysis using mass spectrometry identified approximately 2500 proteins. In three of the HCC patients with post-hepatectomy liver failure (PHLF), 53 proteins were upregulated and 32 proteins were downregulated. The significantly upregulated proteins were DRAP1, GGCT, NSUN2, RAB13, PPCS, and SDHA, and the significantly downregulated proteins were CTSB, TIMM44, VTN, KATNAL2, and RPL27A [46]. Knockdown of lncRNA brain cytoplasmic RNA 1 (BCYRN1) in T24 and BOY bladder cancer cells suppressed proliferation, migration, and invasion. Blood exosomes from 31 bladder cancer patients and 19 healthy controls were collected and quantification of BCYRN1 showed increased expression of BCYRN1 in patients with bladder cancer. In addition, a decrease in BCYRN1 expression was observed in eight patients who underwent complete resection of bladder cancer [47]. Blood samples from 15 ovarian cancer (OC) patients, 14 ovarian cancer patients with anti-Yo-associated paraneoplastic cerebellar degeneration (PCD), and 15 healthy controls were collected and miRNA analysis identified about 100 miRNAs with variable expression. There was a marked upregulation of miR-483-5p, miR-4488, and miR-200c-3p in patients with OC compared to healthy controls. Furthermore, there was a significant upregulation of miR-451a, miR-486-5p, and miR-20b-5p in PCD patients compared to healthy controls, and miR-451a, miR-486-5p, and miR-15a-3p in PCD patients compared to OC patients [48]. Blood exosomes from 134 cervical cancer (CC) patients and 50 healthy controls were collected and lncRNA lymphocytic leukemia deletion gene 1 (DLEU1) was quantified by RT-qPCR. The diagnostic model of DLEU1 showed an AUC of 0.808, sensitivity of 63.4%, and specificity of 86.0%. In the case of CA-125, the AUC was 0.670, sensitivity was 56.0%, and specificity was 87.0%, and in the case of squamous-cell carcinoma (SCC), the AUC was 0.746, sensitivity was 56.7%, and specificity was 93.0%. The combined model of DLEU1, serum CA-125, and SCC showed an AUC of 0.878, sensitivity of 65.7%, and specificity of 94.0% [49]. Exosomes from patients with cholangiocarcinoma, patients suffering from an *O. viverrini* infection that was causing cholangiocarcinoma, and healthy controls were collected, and miRNA analysis by RNA-seq showed that miR-92a-3p, miR-203a-3p, miR-192-5p, miR-223-3p, miR-26a-5p, and miR-194-5p were significantly upregulated in cholangiocarcinoma patients, and in patients infected with O. viverrini, only miR-223-3p was significantly upregulated [50]. Blood exosomes from 30 laryngeal cancer (LCa) patients and 20 healthy controls were collected and exosomal surface proteins were quantified by flow cytometry multiplex analysis. Overexpression of CD1c, CD2, CD3, CD4, CD11c, CD14, CD20, CD44, CD56, CD105, CD146, and CD209 was observed in patients with LCa, while CD24, CD31, and CD40 were not overexpressed in patients with LCa, but were found to be associated with nodal involvement. The diagnostic model of CD56 had the highest AUC of 0.731 in LCa, and the diagnostic model of CD40 had the highest AUC of 0.80 in diagnosis of nodal involvement [51]. The above miRNAs, lncRNAs, and proteins are expected to be used as blood exosome markers for diagnosis.

## 3. Search for Exosome-Derived Cancer Markers in Urine

Urinalysis is very useful in that it can be performed in a noninvasive manner and is less burdensome to the patient. Exosomes are also found in urine, and new biomarkers are being identified from the analysis of cancer-derived urinary exosomes [52] (Table 2).

### 3.1. Urinary Exosomes in Patients with Bladder Cancer

This section describes the latest reports on urinary exosome markers for bladder cancer. RNA-seq of urinary exosomes from three bladder cancer patients and three healthy controls revealed that 145 lncRNAs were upregulated and 13 lncRNAs were downregulated in bladder cancer patients. Among them, RMRP was most significantly upregulated in patients with bladder cancer. Diagnostic testing with RMRP of urinary and plasma exosomes in bladder cancer patients showed an AUC of 0.720 for urine alone, 0.870 for plasma alone, and 0.890 for both combined. RMRP contributed to cancer progression by sponging miR-206 and increasing G6PD expression [53]. RT-RAA-CRISPR/Cas12a technology was developed to detect lncRNA mitochondrial RNA processing endoribonuclease (RMRP) in exosomes in about 30 min. When diagnostic tests were performed on RMRP of urinary exosomes from patients with bladder cancer and healthy controls, and the AUC was 0.946, sensitivity was 0.950, and specificity was 0.943 [54]. Urinary exosomes from 42 bladder cancer patients were collected and quantified for seven lncRNAs (UCA1, H19, MALAT1, TUG1, GAS5, RMRP, and LINC01517), showing increased expression of RMRP, UCA1, and MALAT1 in bladder cancer patients. The diagnostic model was constructed using these genes, and the AUC, sensitivity, and specificity were 0.875, 80.0%, and 81.4%, respectively [55]. Urinary exosomes from 42 bladder cancer patients and 42 healthy controls were collected and quantified for lncRNA SNHG16 by RT-qPCR, showing that lncRNA SNHG16 was highly expressed in bladder cancer patients. When lncRNA SNHG16 was used to construct a diagnostic model, the AUC was 0.791, which was higher than the AUC value (0.597) obtained by urinary cytology [56]. RT-qPCR quantification of miR-146a-5p, miR-93-5p, miR-663b, miR-21, and miR-4454 in urinary exosomes from 116 patients with bladder cancer and 116 healthy controls showed increased expression of these miRNAs in patients with bladder cancer. Furthermore, miR-21 was found to be associated with tumor-node-metastasis staging and grading [57]. Exosomes from 41 non-muscle-invasive bladder cancer patients and 15 healthy controls were collected and subjected to small RNA next-generation sequencing, which revealed 14 differentially expressed transfer RNA-derived fragments (tRFs) (tRF-16-F1R3WEE, tRF-17-8R6546J, tRF-17-I7XUK8N, tRF-17-D9W1X6K, tRF-18-HR1PF7D2, tRF-18-MBQ4NKDJ, tRF-20-40KK5Y93, tRF-21-86J8WPMNB, tRF-25-7P596VW631, tRF-26-IK9NJ4S2I7D, tRF-27-J87383RPD95, tRF-31-PER8YP9LON4VD, tRF-32-PER8YP9LON4V3, and tRF-38-PNR8YP9LON4VN18) [58]. RNA-seq of urinary exosomes from 12 bladder cancer patients and 6 healthy controls identified KLHDC7B as upregulated in bladder cancer patients. High-grade and low-grade bladder urothelial carcinoma (BLCA) could be distinguished by the expression level of KLHDC7B [59]. RT-qPCR quantification of KRT17, GPRC5A, SLC2A1, MDK, and CXCR2 in urinary exosomes from 236 bladder cancer patients and 42 healthy controls showed increased expression of these mRNAs in cancer patients. A diagnostic model of these mRNAs showed AUCs of 0.760 and 0.730, sensitivities of 0.633 and 0.620, and specificities of 0.786 and 0.810 for MDK and KRT17, respectively. KRT17 was significantly upregulated in patients with relapsed disease [60]. RNA-seq analysis of urinary exosomes from 60 bladder cancer patients and 40 healthy controls revealed that 33 genes were upregulated and 156 genes were downregulated in bladder cancer patients. These genes were related to the MAPK pathway, PPAP signaling pathway, PI3K Akt signaling pathway, and Hippo signaling pathway. ROC curves were constructed for tmeff1, SDPR, ACBD7, SCG2, and COL6A2, and the AUC was 0.6934, 0.7746, 0.7239, 0.6396, and 0.6610, respectively. When the ROC curves were constructed for SDPR and acbd7, the AUC was 0.7945, the sensitivity was 89.09%, and the sensitivity was 60.53% [61]. A magnetic 3D ordered macroporous zeolitic imidazolate framework-8 (magMZIF-8), constructed to efficiently separate urinary exosomes, enabled exosome metabolomics analysis using 50 mL of urine, and it took only 2 h to separate exosomes from 42 urine samples. Metabolite analyses in exosomes of bladder cancer patients and healthy controls were performed by LC-MS/MS, showing that arachiconic acid, docosahexaenoic acid, docosapentaenoic acid, and retinyl ester were elevated in bladder cancer patients. When a diagnostic model was constructed by applying machine learning algorithms to the analyzed data, the AUC was 0.875–1.00 [62]. An inorganic (Ti3AlC2)-based exosome collector (MXene@TiO_2_) was developed to collect urinary exosomes from 113 bladder cancer patients and 112 healthy controls, and metabolic profiles were constructed for 465 metabolites using mass spectrometry. The data were used to construct a diagnostic model using machine learning, resulting in an AUC of 0.867 [63]. When urinary exosomes from bladder cancer patients and healthy subjects were collected and analyzed for exosomal glycans, approximately 50 species could be identified, 16 of which showed differential expression. In particular, one upregulated bisecting N-acetylglucosamine (GlcNAc)-type glycan with core fucose, and two upregulated and two downregulated terminal-sialylated glycans were observed in bladder cancer patients [64]. Urinary exosomes from 39 urinary bladder cancer (UBC) patients and healthy controls were collected and analyzed by proximity extension assay, showing that MMP12, MMP7, HO-1, IL8, CD5, CCL20, CXCL13, MCP-1, CD8A, and TGF-beta-1 were upregulated in UBC patients. A model to diagnose muscle invasiveness was constructed from the analyzed data, and the accuracy was 92% [65]. Proteomic data from the urinary exosomes of 261 cancer patients (bladder cancer, prostate cancer, renal cancer, lung cancer, cervical cancer, colorectal cancer, esophageal, and gastric cancer) and 124 healthy controls identified 17 proteins (CD59, CDC42, ITM2B, CD81, PEBP1, VAT1, MYO1D, RAC1, DPP4, RAN, CAPG, PPIA, FOLR1, ANXA3, APOD, ANXA4, and AQP2) important for cancer diagnosis. When a diagnostic model was constructed by machine learning using the expression data of these 17 proteins, the AUC was 0.96 and accuracy was 0.90 [66]. The above miRNAs, lncRNAs, proteins, and metabolites were identified as urinary exosome markers for bladder cancer. It is interesting that metabolites were also detected as markers, and their diagnostic application is expected to increase.

### 3.2. Urinary Exosomes of Prostate Cancer Patients

This section describes the latest reports on urinary exosome markers of prostate cancer. A dumbbell dual-hairpin-triggered DNA nanonet that forms a net structure in the presence of miR-141 was developed to detect miR-141, enabling the detection of miR-141 at 57.6 pM [67]. Urinary exosomes were collected and quantified for miR-451 and miR-21 before and after surgery in 10 PCa patients, showing that both miRNAs were highly expressed preoperatively but low postoperatively [68]. RNA-seq analysis of urinary exosomes from 10 PCa patients and 10 healthy controls, and qRT-PCR analysis of urinary exosomes from 43 PCa patients and 92 healthy controls, revealed PCa patient-specific mRNAs. AUC values were between 0.799 and 0.906 for diagnosis by RAB5B, WWP1, HIST2H2BF, ZFY, MARK2, PASK, RBM10, and NRSN2. Diagnostic modeling with RAB5B and WWP1 showed an AUC of 0.923, 81.4% sensitivity, and 89.1% specificity [69]. Urinary exosomes of prostate cancer (PCa) patients were analyzed with a dual-gate field-effect transistor (DGFET)-based multimarker biosensor, and TMEM256 was upregulated in PCa patients [70]. Urine exosomes from 284 patients who underwent testing to determine prostate cancer were collected and quantified by ELISA for prostate-specific membrane antigen (PSMA), showing that PSMA was upregulated in PCa patients compared to benign patients, with an AUC of 0.876 [71]. In PCa patients, cancer-cell-derived exosomal PSM-E is upregulated in the serum and urine. When exosomal PSM-E is incorporated into the M0 macrophage, PSM-E binds to the fourth tryptophan aspartate repeat of RACK1 in the protease-associated domain and suppresses FAK and ERK signaling pathways, thereby inhibiting M2 macrophage polarization, resulting in the suppression of prostate cancer cell proliferation, invasion, and metastasis [72]. Urinary exosomes were collected from 272 prostate biopsy patients, and urinary exosomal prostate-specific antigen (UE-PSA) was quantified by ELISA, showing that increased expression of UE-PSA was observed in PCa patients compared to benign patients with an AUC of 0.953 [73]. The above miRNAs and proteins are expected to be used as urinary exosome markers for the diagnosis of prostate cancer.

### 3.3. Urinary Exosomes of Other Cancer Patients

This section describes the latest reports on urinary exosome markers for cancers other than those described in the previous sections. When exosomes were collected from the urine of 100 lung cancer patients and 100 healthy controls by nanowire, approximately 2500 miRNAs were detected, 48 of which were upregulated (miR-1250-5p, miR-1254, miR-1273f, miR-1910-3p, miR-3064-3p, miR-3164, miR-3591-3p, miR-3691-5p, miR-424-3p, miR-4296, miR-4300, miR-4306, miR-4311, miR-4321, miR-4428, miR-4429, miR-4436b-3p miR-4453, miR-4470, miR-4520-3p, miR-4520-5p, miR-4525, miR-4538, miR-4644, miR-4647, miR-4657, miR-4660, miR-4692, miR-4727-3p, miR-4784, miR-5093, miR-5189-5p, miR-551b-5p, miR-5698, miR-6076, miR-6131, miR-614, miR-650, miR-6515-5p, miR-6747-5p, miR-6760-5p, miR-6801-5p, miR-6815-5 p, miR-6828-5p, miR-7151-3p, miR-766-5p, miR-8057, and miR-921) and 6 of which were downregulated (miR-20a-3p, miR-374c-3c, miR-431-5p, miR-452-3p, miR-642a-3p, and miR-671-5p) in lung cancer patients. The upregulated miRNAs included those related to the MAPK signaling pathway and the PI3K-Akt signaling pathway. Furthermore, when analyzed data were applied to machine learning-based analysis to construct a diagnostic model, the AUC was 0.99 [74]. Through LC-MS/MS analysis of urinary exosomes from 30 healthy controls, 12 lymphocyte migration regulation-related proteins (WASL, STK10, SPNS2, STK10, PKD1, LCK, and GP2) were identified. Among them, WASL, STK10, and WNK1 were diagnosed in the urinary exosomes of 44 patients with lung cancer, with an AUC of 0.760 [75]. Urinary exosomes from nine pancreatic ductal adenocarcinoma (PDAC) patients and seven healthy controls were collected, and miRNA analysis showed that the miR-3940-5p/miR-8069 ratio was increased in PDAC patients. When the diagnosis was made using the miR-3940-5p/miR-8069 ratio and CA19-9, the sensitivity was 93.0% and the positive predictive value was 78.4% [76]. Exosomes were recovered from urine of five pancreatic cancer patients and five healthy controls using phosphatidylserine molecularly imprinted polymers (PS-MIPs), and proteomic analysis by mass spectrometry showed that SLC9A 3R1, SPAG9, and ferritin light chain (FTL) were upregulated in pancreatic cancer patients, suggesting their potential diagnostic markers for pancreatic cancer [77]. Mass spectrometry analysis of urinary exosomes from 10 epithelial ovarian cancer (EOC) patients and 10 healthy controls revealed increased expression of leucine-rich alpha-2-glycoprotein 1 (LRG1) in EOC patients. LRG1 expression is associated with poor prognosis and activates the focal adhesion kinase/protein kinase B (FAK/AKT) signaling pathway, which promotes cancer progression [78]. Urinary exosomes from four ovarian cancer patients and two healthy controls were collected and quantified for CD117. CD117 was not detected in healthy controls, but in the case of patients with high-grade serous ovarian carcinoma and papillary serous cystadenocarcinoma, CD117 was upregulated [79]. To rapidly detect miRNAs in exosomes, fusogenic nanoreactor (FNR)-encapsulating DNA-fueled molecular machines (DMMs) were constructed and miR-222, miR-200c, and miR-375 were quantified in the exosomes of breast cancer patients and healthy controls. Increased expression of these miRNAs was observed in breast cancer patients. In addition, a discrimination test between breast cancer patients and healthy controls was performed, and the diagnostic accuracy was 86.4% [80]. Surface-enhanced Raman spectroscopy (SERS) was performed to detect exosomal miRNAs, and it was able to detect exosomal miRNAs at concentrations as low as 0.5 nM. When exosomal miRNAs of cancer cells and normal cells were analyzed, exosomes derived from cancer cells were richer in miRNAs. In addition, exosomal miRNA levels were found to increase when cells were subjected to electrical stimulation [81]. miRNA-seq was performed on urinary exosomes from renal-cell carcinoma patients and healthy controls, and an increased expression of miR-542-5p and decreased expression of miR-320a were observed in cancer patients [82]. RNA-seq of urinary exosomes from 47 clear-cell renal-cell carcinoma (ccRCC) patients and 16 urolithiasis controls revealed that in ccRCC patients, SNORD99, SNORD22, SNORD26, and SNORA50C were downregulated in ccRCC patients. When a diagnostic model was constructed using SNORD99, SNORA50C, obesity, and hypertension, the accuracy was 0.811 [83]. Urinary exosomes from 30 Wilms’ tumor patients with a rare kidney cancer and 27 healthy controls were collected and quantified by qPCR for metastasis-associated lung adenocarcinoma transcript-1 (MALAT1), showing that MALAT1 expression was decreased in Wilms’ tumor patients [84]. PD-L1 and Alix were quantified in the urinary exosomes of 26 urothelial cancer patients and 12 healthy controls by ELISA and it was found that the expression of PD-L1 and Alix tended to be elevated in cancer patients [85]. Proteomic analysis of urinary exosomes from nine CRC patients and three healthy controls using label-free liquid chromatography–tandem mass spectrometry (LC-MS/MS) revealed the upregulation of 67 proteins and downregulation of 74 proteins in CRC patients. In particular, increased expression of CEACAM7 and CEACAM1, and decreased expression of CHMP4A, CHMP4B, CHMP2A, CHMP2B, and CHMP1B, were prominent in CRC patients [86]. When urinary exosomes from 21 patients with thyroid carcinoma were collected and analyzed for proteins by mass spectrometry, increased expression of tissue inhibitors of metalloproteinases (TIMPs) was observed in patients with lymph node metastasis [87]. Proteomic analysis of urinary exosomes from 18 hepatocellular carcinoma patients and healthy controls by mass spectrometry revealed increased expression of OLFM4, HDGF, and GDF15 in patients with hepatocellular carcinoma [88]. The above RNAs and proteins were identified as urinary exosome markers. It is interesting to note that changes were also observed in the spectral analysis of exosomes, which is expected to have diagnostic applications.

## 4. Conclusions and Future Directions

As described above, analysis of exosome contents in blood and urine has identified proteins, miRNAs, lncRNAs, and mRNAs that are promising cancer markers. It is interesting to note that the molecular species detected in the analysis of exosome contents differ depending on the report, even for the same cancer type. This may be due to differences in extraction methods, analysis methods, and sample storage methods. Therefore, it may be necessary to consider these conditions when applying exosomes to diagnosis. Since blood and urine samples are less burdensome for patients, diagnosis by blood and urine exosomes is a simple test for patients. Thus, regular testing may facilitate early detection of cancer. Exosomes are released from many cells, so ensuring the specificity of the marker will be a challenge in the future. If the issue of specificity in exosome diagnostics can be overcome, more cancer types could be addressed. In particular, its application to cancers that are difficult to detect at an early stage, such as pancreatic cancer, is highly promising. miR-21 is deeply involved in cancer progression in pancreatic cancer, and its use as a marker may be possible [89]. Exosomes contain information about cancer. Therefore, by analyzing exosomes, we may be able to learn the characteristics of cancer and use them not only for diagnosis but also for treatment selection. The use of exosomes may be cost-effective in that some information that cannot normally be obtained without resection of the cancer and analysis of the cancer tissue can be obtained by analysis of exosomes. Further analysis will continue in the future to achieve higher accuracy in cancer diagnosis. The discovery of new cancer markers through machine learning is particularly remarkable. As AI technology improves and the computational power of computers increases, it is expected that new indicators that have not been noticed by the human eye will become increasingly apparent. In addition, the improved capabilities of analyzers will yield big data. It will become possible to construct a system that can perform multifaceted cancer diagnosis by integrating big data obtained from patients and information on cancer markers identified so far. Such a system may already exist in certain organisms. In recent years, cancer diagnosis has become possible with nematodes [90]. Nematodes have hundreds of olfactory receptors that can detect cancer-derived substances in urine and the detection appears as chemotaxis [91]. In other words, they can answer the question of whether cancer is present or not from a large amount of input information. It is important to utilize this ability. RNA-seq technology has made it possible to analyze various RNA molecules. Recently, RNA modifications such as m^6^A have been found to be important for cancer growth [92,93]. Furthermore, it has been reported that modified RNA molecules are also present in exosomes, so it is likely that more and more modified RNA molecules will be found as cancer markers in the future as the measurement technology of modified RNA molecules improves [94]. In addition, since lncRNAs have recently been found to encode micropeptides, they may also be potential cancer marker candidates [95,96,97]. Surely a bright future awaits us in the field of cancer biomarker discovery.

## Figures and Tables

**Table 1:** Cancer marker candidates in blood exosomes.

Candidate Cancer Markers	Molecular Types	Isolation Methods	Analysis Methods	Cancer Types	References
piR-36,340, piR-33,161, miR-484, miR-548ah-5p, miR-4282, and miR-6853-3p	piRNA or miRNA	exoEasy maxi kit (QIAGEN, Hilden, Germany)	RT-qPCR	Breast	[19]
miR-200c	miRNA	Hhieff^®^ quick exosome isolation kit (YEASEN, Shanghai, China)	RT-qPCR	Breast	[20]
miR-6831-5P	miRNA	Exosome rapid extraction reagent kit (YEASEN, Shanghai, China)	RT-qPCR	Breast	[21]
CEA, CA125, and EGFR	Glycoprotein/protein	Integrated centrifugal disk chip	ELISA	Breast	[22]
CD9 and Her2	Protein	Antibody-conjugated disk	ELISA	Breast	[23]
PD-L1, EpCAM, and EGFR	Protein	DEP–ELISA chip	ELISA	Breast, colon, and lung	[24]
miR-21-5p, miR-126-3p, miR-210-3p, miR-221-3p, Let-7b-5p, miR-146a-5p, miR-222-3p, and miR-9-5p	miRNA	exoEasy maxi kit (QIAGEN, Hilden, Germany)	RT-qPCR	Lung	[25]
miR-29a-3p	miRNA	Macherey-Nagel™ exosome precipitation solution for serum/plasma (Fisher Scientific, Waltham, MA, USA)	RT-qPCR	Lung	[26]
SNORD116 and SNORA21	snRNA	Ultracentrifugation	Microarray	Lung	[27]
circ-0033861, circ-0043273, and circ-0011959	circRNA	Ultracentrifugation	Microarray	Lung	[28]
CXCL12, TFBR2, CD44v6, HIF1A, and KRT7	mRNA	Total exosome isolation kit (Invitogen, Carlsbad, CA, USA)	RT-qPCR	Lung	[29]
EGFR mutations	DNA	XCF^TM^ Exosomal DNA isolation kit (System Biosciences, Palo Alto, CA, USA)	RT-qPCR	Lung	[30]
c-Myc, Snail, MAVS, and STING	Protein	Ultracentrifugation	Western blot	Lung	[31]
Raman spectrum	Exosome	Ultracentrifugation	Raman spectrum	Lung	[32]
TF-Ag-α	Glycoprotein	Ultracentrifugation	Surface plasmon resonance (SPR)	Lung and breast	[33]
LINC01268, LINC02802, AC124854.1, and AL132657.1	lncRNA	exoRNeasy midi kit (QIAGEN, Hilden, Germany)	RNA-seq	Pancrea	[34]
miR-6855-5p	miRNA	qEVTM original 35 nm size exclusion column (Izon Science, Christchurch, New Zealand)	RNA-seq	Pancrea	[35]
miR-21, miR-191, and miR-451a	miRNA	ExoQuick (System Biosciences, Palo Alto, CA, USA)	RT-qPCR	Pancrea	[36]
miR-6891-5p, miR-6732-5p, and miR-1234-3p	miRNA	qEVTM original 35 nm size exclusion column (Izon Science, Christchurch, New Zealand)	Microarray	Pancrea	[37]
ARNTL2, FHL2, KRT19, MMP1, CDCA5, and KIF11	mRNA	ExoRBase 2.0 database	RNA-seq	Pancrea	[38]
lncRNA NAMPT-AS	lncRNA	Exosome isolation kit (Miltenyi Biotec, Bergisch Gladbach, Germany)	RNA-seq	Colon	[39]
miR-425–5 p, Let-7f-5p, C19orf43, TOP1, PPDPF, LNC-EV-9572, lnc-MKRN2-42:1, HIST2H2AA4, and MT-ND2	miRNA, mRNA, and lncRNA	Ultracentrifugation	RNA-seq	Colon	[40]
5-methylcytosine miRNA-21	Modified miRNA	Ultracentrifugation	DNAzyme-triggered rolling circle amplification	Colon	[41]
PF4 and AACT	Protein	Ultracentrifugation	ELISA	Colon	[42]
miR-21-5p, miR-320, miR-191-5p, and miR-451	miRNA	Total exosome isolation kit (Thermo Fisher Scientific, Waltham, MA, USA)	RNA-seq	Gastric	[43]
MUC1	Protein	Ultracentrifugation	Raman spectrum	Gastric	[44]
TRIB3 and NQO1	mRNA	Ultracentrifugation	RNA-seq	Hepatocellular carcinoma	[45]
DRAP1, GGCT, NSUN2, RAB13, PPCS, SDHA, CTSB, TIMM44, VTN, KATNAL2, and RPL27A	Protein	Ultracentrifugation	Mass spectrometry	Hepatocellular carcinoma	[46]
lncRNA brain cytoplasmic RNA 1 (BCYRN1)	lncRNA	exoEasy maxi Kit (QIAGEN, Hilden, Germany)	RNA-seq	Bladder	[47]
miR-483-5p, miR-4488, and miR-200c-3p	miRNA	exoRNeasy midi kit (QIAGEN, Hilden, Germany)	RNA-seq	Ovarian	[48]
lncRNA DLEU1	lncRNA	Ultracentrifugation	RT-qPCR	Cervical	[49]
miR-92a-3p, miR-203a-3p, miR-192–5p, miR-223–3p, miR-26a-5p, and miR-194–5p	miRNA	exoRNeasy serum/plasma midi kit (QIAGEN, Hilden, Germany)	RNA-seq	Cholangiocarcinoma	[50]
CD1c, CD2, CD3, CD4, CD11c, CD14, CD20, CD44, CD56, CD105, CD146, and CD209	Protein	Exosome isolation kit (Miltenyi Biotec, Bergisch Gladbach, Germany)	Flow cytometry	Laryngeal	[51]

**Table 2:** Cancer marker candidates in urine exosomes.

Candidate Cancer Markers	Molecular Types	Isolation Methods	Analysis Methods	Cancer Types	References
lncRNA RMRP	lncRNA	ExoQuick kit (Bestbio, Shanghai, China)	RT-qPCR	Bladder	[53,54]
lncRNA RMRP, UCA1, and MALAT1	lncRNA	Ultracentrifugation	RT-qPCR	Bladder	[55]
lncRNA SNHG16	lncRNA	Exosome RNA isolation kit (Rengen Biosciences, Shenyang, China)	RT-qPCR	Bladder	[56]
miR-21	miRNA	Ultracentrifugation	RT-qPCR	Bladder	[57]
tRF-16-F1R3WEE, tRF-17-8R6546J, tRF-17-I7XUK8N, tRF-17-D9W1X6K, tRF-18-HR1PF7D2, tRF-18-MBQ4NKDJ, tRF-20-40KK5Y93, tRF-21-86J8WPMNB, tRF-25-7P596VW631, tRF-26-IK9NJ4S2I7D, tRF-27-J87383RPD95, tRF-31-PER8YP9LON4VD, tRF-32-PER8YP9LON4V3, and tRF-38-PNR8YP9LON4VN18	Transfer RNA-derived fragment	exoRNeasy kit (QIAGEN, Hilden, Germany)	RNA-seq	Bladder	[58]
KLHDC7B	mRNA	Ultracentrifugation	RT-qPCR	Bladder	[59]
KRT17, GPRC5A, SLC2A1, MDK, and CXCR2	mRNA	ExoComplete tube kit (Showa Denko Materials, Tokyo, Japan)	RT-qPCR	Bladder	[60]
tmeff1, SDPR, ACBD7, SCG2, and COL6A2	mRNA	Ultracentrifugation	RNA-seq	Bladder	[61]
Arachiconic acid, docosahexaenoic acid, docosapentaenoic acid, and retinyl ester	Metabolite	magMZIF-8	Mass spectrometry	Bladder	[62]
Metabolic profiles	Metabolite	MXene@TiO_2_/Fe_3_O	Mass spectrometry	Bladder	[63]
Glycans	Sugar	Ultracentrifugation	Mass spectrometry	Bladder	[64]
MMP12, MMP7, HO-1, IL8, CD5, CCL20, CXCL13, MCP-1, CD8A, and TGF-beta-1	Protein	Ultracentrifugation	Flow cytometry	Bladder	[65]
CD59, CDC42, ITM2B, CD81, PEBP1, VAT1, MYO1D, RAC1, DPP4, RAN, CAPG, PPIA, FOLR1, ANXA3, APOD, ANXA4, and AQP2	Protein	Public exosome proteomics data	Mass spectrometry	Bladder, prostate, renal, lung, cervical, colorectal, esophageal and gastric	[66]
miR-141	miRNA	exoEasy maxi kit (QIAGEN, Hilden, Germany)	DNA nanonet	Prostate	[67]
miR-451 and miR-21	miRNA	Urine microRNA purification kit (Norgen Biotek, Thorold, ON, Canada)	ssDNA sensor	Prostate	[68]
RAB5B, WWP1, HIST2H2BF, ZFY, MARK2, PASK, RBM10, and NRSN2	mRNA	Ultracentrifugation	RNA-seq	Prostate	[69]
TMEM256	Protein	ExoQuick-TC™ (System Biosciences, Palo Alto, CA, USA)	ELISA	Prostate	[70]
PSMA	Protein	Exosome isolation kit (Wayen, Shanghai, China)	ELISA	Prostate	[71]
PSM-E	Protein	Ultracentrifugation	Mass spectrometry	Prostate	[72]
Urinary exosomal prostate-specific antigen (UE-PSA)	Glycoprotein	Exosome isolation kit (Wayen, Shanghai, China)	ELISA	Prostate	[73]
54 miRNAs	miRNA	ZnO nanowires	Microarray	Lung	[74]
CX3CL1,WNK1, GBA, CD58, WASL, LGALS8, MSN, SPNS2, STK10, PKD1, LCK, and GP2	Protein	Ultracentrifugation	Mass spectrometry	Lung	[75]
miR-3940-5p/miR-8069	miRNA	ExoQuick-TC (System Biosciences, Palo Alto, CA, USA)	Microarray	Pancrea	[76]
SLC9A3R1, SPAG9, and ferritin light chain (FTL)	Protein	Phosphatidylserine molecularly imprinted polymers	Mass spectrometry	Pancrea	[77]
Leucine-rich alpha-2-glycoprotein 1(LRG1)	Glycoprotein	Ultracentrifugation	Mass spectrometry	Ovarian	[78]
CD117	Protein	100 kDa Amicon Ultra-15 centrifugal filter units (Millipore, Boston, MA, USA)	Flow cytometry	Ovarian	[79]
miR-222, miR-200c, and miR-375	miRNA	miRNeasy serum/plasma kit (QIAGEN, Hilden, Germany)	RT-qPCR	Breast	[80]
Raman spectrum	Exosome	Surface-enhanced Raman spectroscopy	Raman spectrum	MCF-7, HeLa, and H8 cell lines	[81]
miR-542-5p and miR-320a	miRNA	Exo-Urine™ EV isolation kit (System Biosciences, Palo Alto, CA, USA)	RNA-seq	Renal	[82]
SNORD99 and SNORA50C	snRNA	miRCURY exosome cell/urine/CSF kit (QIAGEN, Hilden, Germany)	RNA-seq	Renal	[83]
lncRNA MALAT1	lncRNA	Total exosome isolation kit (Invitogen, Carlsbad, CA, USA)	RT-qPCR	Wilms’ tumor (a rare kidney cancer)	[84]
PD-L1 and Alix	Protein	ExoDisc (LabSpinner, San Diego, CA, USA)	ELISA	Urothelial	[85]
CEACAM7, CEACAM1, CHMP4A, CHMP4B, CHMP2A, CHMP2B, and CHMP1B	Protein	Ultracentrifugation	Mass spectrometry	Colorectal	[86]
TIMP	Protein	ExoQuick-TC (System Biosciences, Palo Alto, CA, USA)	Mass spectrometry	Thyroid	[87]
OLFM4, HDGF, and GDF15	Protein	Array-based amphiphilic supramolecular probe (ADSP)-modified membranes	ELISA	Hepatocellular	[88]

## Data Availability

Not applicable.
